# A Simple Microfluidic Device to Mitigate the Effect of Faradaic Reactions in Cross-Stream Particle Migration in DC-Electrokinetics

**DOI:** 10.3390/mi17020248

**Published:** 2026-02-13

**Authors:** Juan Arcenegui-Troya, Pablo García-Sánchez, Antonio Ramos

**Affiliations:** Departamento Electrónica y Electromagnetismo, Facultad de Física, Universidad de Sevilla, Avenida Reina Mercedes s/n, 41012 Sevilla, Spain; pablogarcia@us.es (P.G.-S.); ramos@us.es (A.R.)

**Keywords:** electrokinetics, electrophoresis, faradaic reactions, microfluidics

## Abstract

Direct-current (DC) electrokinetics in microfluidic channels is inherently affected by Faradaic reactions at the electrode–electrolyte interfaces, which induce local changes in pH and conductivity and, consequently, alter particle behavior. In this work, we present a simple microfluidic T-junction device designed to mitigate these effects by continuously flushing the regions near the electrodes with fresh electrolyte, thereby preserving the physicochemical properties of the main channel. Using fluorescence imaging with a pH-sensitive dye and electrical resistance measurements, we demonstrate that electrolyte acidification caused by water electrolysis can be effectively suppressed when advection overcomes electromigration of H^+^ ions. Order-of-magnitude estimates based on ion transport reveal that this condition is achieved when the flow velocity exceeds the characteristic electromigration velocity. We further investigate the effect of Faradaic reactions on cross-stream particle migration in electrophoresis experiments by quantifying the separation between suspended particles and the channel walls. We find that the particle–wall separation is significantly larger when electrolyte modifications are suppressed, clearly demonstrating the influence of Faradaic reactions on this phenomenon. Our results show that minimizing electrolyte modifications leads to a significantly enhanced particle-wall separation, highlighting the strong influence of Faradaic reactions on electrokinetic outcomes. These findings emphasize the importance of controlling electrochemical effects in DC electrokinetics and provide a simple and robust strategy to improve the accuracy and reproducibility of microfluidic electrophoresis experiments.

## 1. Introduction

Electric fields are often employed to drive the motion of electrolytes and/or particles dispersed in liquids—a research field known as electrokinetics. A well-known case of electrokinetic phenomenon is electrophoresis, which refers to the motion of a particle through an electrolyte when an external electric field interacts with the electrical charges present in the electric double layer (EDL) at the particle–electrolyte boundary [1,2]. This phenomenon is widely used to separate and analyze colloidal particles [3], as well as large molecules like DNA and proteins [4]. More broadly, electrophoresis applies to any particle that acquires a net surface charge when dispersed in an electrolyte. The electrophoretic velocity is related to the zeta potential ζ of a particle through the Helmholtz–Smoluchowski equation [1]: $u_{\mathrm{ep}}=\zeta \varepsilon E/\eta$, where *E* is the magnitude of the applied electric field, and ε and η represent the electrical permittivity and viscosity of the liquid, respectively.

Electrokinetic phenomena in microfluidic channels are commonly studied by inserting metal electrodes at the ends of the microchannel [5,6,7,8,9,10,11,12]. Applying a voltage difference between the electrodes generates an electric field within the channel. Because the electrolyte behaves as an ohmic conductor, the presence of an electric field implies an electrical current flowing through the channel. Since the charge carriers are electrons in metals and ions in the electrolyte, current continuity implies charge transfer reactions at the electrode-electrolyte interfaces, also known as Faradaic reactions. As a result of the Faradaic reactions at the electrodes the composition of the electrolyte is altered, leading to local changes in conductivity and/or pH [13,14,15,16,17,18].

These electrolyte modifications have two major consequences for the electrophoretic behavior of suspended particles. First, the particle zeta potential generally depends on both the pH and the ionic strength of the solution [19,20]. Second, spatial variations in electrolyte conductivity lead to corresponding variations in the electric field along the microchannel. This latter effect follows directly from current conservation along the channel. Besides the effect on the electrophoretic motion, these electrolyte modifications might influence cross-stream particle migration experiments [21], where collinear electric field and pressure-driven flow are combined in microchannels and, as a result, particles drift perpendicular to the direction of the electrophoretic motion, i.e., perpendicular to the direction of the electric field. For example, experiments with microparticles in refs. [21,22,23] report particle migration either toward or away from the microchannel walls, depending on whether the electric and velocity fields are parallel or antiparallel. Although experimental observations have been attributed to the Saffman inertial-lift effect [24], the underlying mechanism behind this phenomenon is far from clear and currently under investigation, also for its potential application in fractionation of micron-sized particles [21].

In this paper, we propose a simple microfluidic device designed to mitigate the impact of Faradaic currents on electrokinetics experiments with applied DC voltages, thereby avoiding or minimizing the electrolyte property changes described above. The device employs a T-junction configuration in which fresh electrolyte is continuously injected, while the fluid in the vicinity of the electrodes is flushed away. As a result, the main channel remains filled with electrolyte whose physicochemical properties are preserved. To demonstrate the effectiveness of this approach, we combine electrical measurements with fluorescence imaging using a pH-sensitive dye, showing that the electrolyte properties in the main channel remain unchanged. This behavior is in sharp contrast with that observed in a single-channel configuration, where Faradaic reactions lead to significant local modifications of the electrolyte. Importantly, we study the effect of Faradaic reactions on cross-stream particle migration experiments by quantifying the separation between suspended particles and the channel walls. We find that the particle–wall separation is significantly larger when electrolyte modifications are suppressed, clearly demonstrating the influence of Faradaic reactions on this phenomenon.

## 2. Methodology

**Figure 1:**
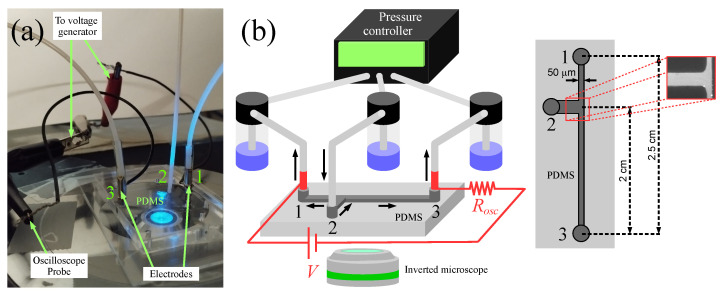
Photograph (**a**) and schematic representation (**b**) of the experimental setup (not to scale). In (**b**), the schematic of the microfluidic channel with dimensions is shown, along with an experimental image of the channel junction.

Figure 1a,b show, respectively, a photograph and a schematic of the experimental setup. The microfluidic device, fabricated using standard soft lithography techniques, consists of a PDMS microchannel with three inlets (labeled 1, 2, and 3 in Figure 1). The device comprises two main channels that intersect at a T-shape junction. The shorter channel is 2 mm long with a rectangular cross-section measuring 165 μm in width and 80 μm in height. The longer channel (main channel) is 25 mm long, 50 μm wide, and also 80 μm high. An electric field was applied along the longer channel by imposing a 2000 V potential difference between the metal needles placed in inlets 1 and 3. Flow rate and direction of the fluid flow were controlled using a pressure controller connected to liquid reservoirs via plastic tubings. An electrolyte solution was prepared by dissolving KCl in deionized (DI) water to obtain a concentration of 0.1 mM. The electrical conductivity of the solution was measured using a conductivity meter (LF3000, WTW TM, Weilheim, Germany) equipped with a probe designed for aqueous solutions. The measured conductivity was 1.45 mS/m. The electric current *I* in the channel was monitored by connecting an oscilloscope probe to inlet 3, as shown in Figure 1b. By Ohm’s law, $I=V_{\mathrm{osc}}/R_{\mathrm{osc}}$, where $R_{osc}=1\;M\Omega$ is the resistance of the oscilloscope probe and $V_{\mathrm{osc}}$ the voltage measured by the oscilloscope. Since $R_{osc}$ is much smaller than the estimated electrical resistance of the 2.5 mm-long channel segment, ∼5 GΩ, it follows that the channel resistance is obtained as $R_{\mathrm{CH}}\approx V/I=VR_{\mathrm{osc}}/V_{\mathrm{osc}}$.

Variations in solution pH were monitored using a pH-sensitive fluorescent dye (fluorescein puriss, Riedel-de Haën TM, Seelze, Germany), for which a decrease in fluorescence intensity indicates a more acidic electrolyte. Carboxylate Fluorescent latex particles (3 μm diameter) were dispersed in the electrolyte (Polysciences, Inc., Warrington, PA, USA). Their zeta-potential was measured using a Zetasizer Nano ZS (Malvern Panalytical Ltd., Malvern, UK, resulting in −81 mV. These particles were used as flow tracers when no electric fields were applied, and exhibited electrophoretic motion upon application of the electric field. Images of the junction were acquired using a high-sensitivity camera (ORCA-R2, Hamamatsu Photonics KK, Hamamatsu, Japan) mounted on an inverted fluorescence microscope, with a 20X objective focused at the mid-height of the channel cross-section.

**Figure 2:**
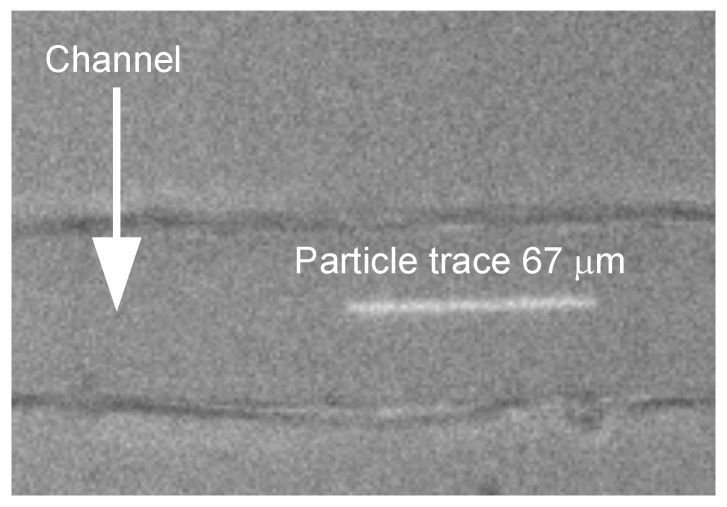
Example of a frame used to measure the particle velocity. The exposure time in this case was 1 ms and, therefore, the particle velocity is 67 mm/s.

The liquid velocity at the center of the channel was determined from the trace left by the fluorescent latex particles over a given time interval corresponding to the camera exposure time (1 ms), namely, velocity=tracelength/exposuretime. Each value was calculated as the average of ten particle traces. Figure 2 shows an example of a frame used to measure the velocity.

## 3. Results and Discussion

In this section, we present the results of our experiments using fluorescent dyes and particles, together with measurements of the electrical resistance of the channel. These results are discussed in the context of the effects of Faradaic currents on the liquid properties and on the cross-stream particle migration in electrophoresis experiments.

### 3.1. Experiments with Fluorescein

**Figure 3:**
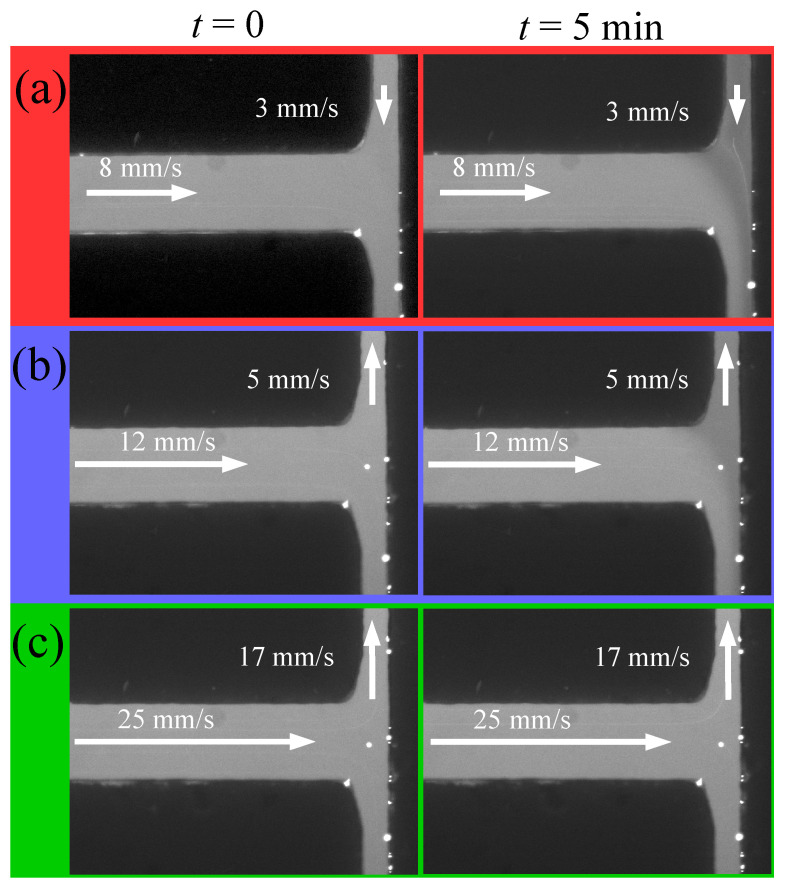
Images of the channel intersection captured under various experimental conditions, with different flow rates and flow directions in the channel connecting reservoirs 1 and 3: (**a**) 3 mm/s toward inlet 3, (**b**) 5 mm/s toward inlet 1, and (**c**) 17 mm/s toward inlet 1. Flow direction and velocity are indicated by arrows. The left column shows the state before activation of the voltage supply, whereas the right column presents the state after five minutes of applied voltage.

Figure 3 shows fluorescence images of the microfluidic junction filled with an electrolyte containing fluorescein, acquired at different fluid velocities as indicated. The white arrows indicate the direction of the flow. The first column of three images on the left corresponds to the initial state, prior to activating the voltage supply, while the column on the right shows the state after the voltage had been applied for five minutes. For *t* = 0, the fluorescein brightness is homogeneous at the channel intersection, and no spatial variations in pH are observed. After five minutes, cases (a) and (b) exhibit a distinct behavior, characterized by the emergence of a darker region in the longer channel. In the experiments, the darker region consistently advanced from inlet 1 toward inlet 3, and never in the opposite direction. This change in fluorescence intensity indicates that the pH decreases near the electrode in inlet 1, that is, the liquid near the anode becomes more acidic.

According to several authors [12,25], water electrolysis can occur under two distinct scenarios depending on the pH of the electrolyte, both of which lead to increased acidity near the anode (located at inlet 1), in agreement with our observations. In contrast, as observed in case (c), the formation of the dark region can be suppressed by increasing the flow velocity. This behavior can be rationalized by considering the flux of a chemical species within the electrolyte:

(1) $$J=-D\nabla c+cv+\frac{Dq}{k_{B}T}cE,$$

where *D* is the diffusion coefficient, *c* is the concentration, v is the fluid velocity, *q* is the electrical charge, $k_{B}$ is the Boltzmann constant, *T* is the absolute temperature and E is the electric field. The first term on the right-hand side of Equation (1) represents diffusion, the second term corresponds to advection, and the third term accounts for electromigration. Under our experimental conditions, with a flow velocity of *v*∼$10^{-3}$ m/s, a diffusion coefficient for H^+^ of $D_{H^{+}}$ = 9.1·10^−9^ m^2^/s [26] and a characteristic length from the intersection to inlet 1 of *L* = 5·10^−3^ m, the Péclet number is $Pe=Lv/D_{H^{+}}=550$, indicating that advection dominates over diffusion. It is also possible to compare the relative contributions of diffusion and electromigration. According to Equation (1), the electromigration velocity is given by $v_{em}=D_{H^{+}}eE/k_{B}T=28$ mm/s. Drawing an analogy with the concept of the Péclet number, we can define the dimensionless ratio of electromigration to diffusion as $Lv_{em}/D_{H^{+}}=15400$, which reveals that electromigration dominates over diffusion. The electric field is estimated by dividing the applied voltage by the total length of the channel connecting inlets 1 and 3: $E=8\cdot 10^{4}$ V/m. Therefore, the electromigration velocity, $v_{em}=28$ mm/s, is comparable to the advection velocity in the channel connecting the intersection to inlet 1.

In light of this calculation, the scenarios shown in Figure 3 become easier to interpret. Advection prevents H^+^ ions from entering the channel when its direction opposes that of the applied electric field. In contrast, electromigration consistently drives H^+^ ions into the channel. When the advection is aligned with the direction of the electric field, as shown in Figure 3a), it also contributes to driving H^+^ ions into the channel. This results in a darker region, indicating a decrease in pH. When advection opposes electromigration, its velocity must exceed the electromigration velocity to completely prevent H^+^ ions from entering the channel. Therefore, our order of magnitude estimates require that the advection velocity has to be of the order or larger than 28 mm/s. According to the scenario represented in Figure 3c, at a advection velocity of 17 mm/s, the presence of H^+^ cannot be detected by a change in brightness.

### 3.2. Electrical Measurements

**Figure 4:**
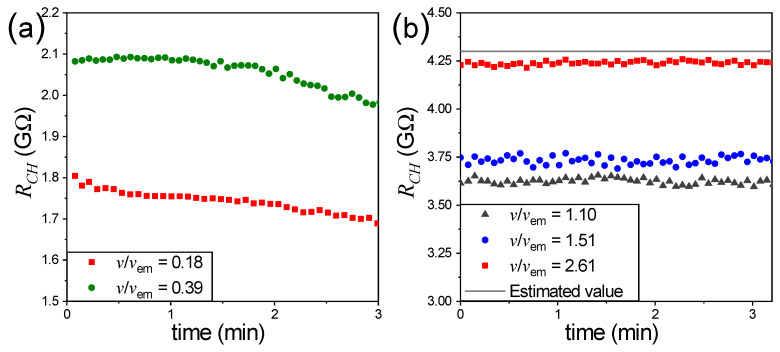
Time evolution of $R_{\mathrm{CH}}$ for different advection velocities measured at the center of the channel connecting the intersection to inlet 1, with the flow opposing the direction of the electric field. The velocities are expressed relative to the electromigration velocity. (**a**) Cases where $R_{\mathrm{CH}}$ decreases with time. (**b**) Cases where $R_{\mathrm{CH}}$ remains steady.

The electrical resistance of the channel was determined by measuring the electric current flowing through the channel. To this end, we used a resistor *R* in series with the channel—the current through the channel also flows through the resistor. As explained above, the voltage drop across the oscilloscope probe allows us to determine the current *I*, which relates to the applied voltage V=2000 V through the electrical resistance of the channel $R_{\mathrm{CH}}=V/I$. Figure 4 shows the time evolution of $R_{\mathrm{CH}}$ for different advection velocities measured at the center of the channel connecting the intersection to inlet 1, with the flow opposing the direction of the electric field. The velocities are expressed relative to the electromigration velocity, $v_{em}=28$ mm/s. We distinguish between two situations: Data in Figure 4a show that $R_{\mathrm{CH}}$ decreases over time, whereas in those shown in Figure 4b, $R_{\mathrm{CH}}$ remains steady. The electric resistance of a channel with length *l*, cross section *S*, and conductivity σ is given by $R_{\mathrm{CH}}=l/S\sigma$. Therefore, a decrease in resistance over time indicates an increase in conductivity, consistent with the fact that advection does not fully counteract electromigration, leading to a rise in the concentration of H^+^ ions. When the advection velocity is comparable to the electromigration velocity, as in the cases represented by the blue circles and dark grey triangles in Figure 4b, the channel resistance remains steady. However, it does not reach the expected value corresponding to a conductivity of σ=1.45 mS/m, which is the measured conductivity of the KCl solution used. This may be attributed to the fact that fluid flow in a channel with a rectangular cross-section exhibits a non-uniform velocity profile, with a maximum at the center of the channel and a minimum (zero) velocity near the walls. Thus, while the flow velocity exceeds electromigration in the center of the channels, electromigration still dominates in other regions of the cross-section. For a centerline flow velocity of $v=2.61v_{em}$, $R_{\mathrm{CH}}$ nearly reaches its expected value and, therefore, under these conditions, the concentration of H^+^ ions in the channel is minimal. Notice that we have not considered the contribution of conductance on the surface of the channel walls [27,28], which plays an important role in nanochannels [29]. The relative influence of surface conductance is commonly given by the Dukhin number (Du), defined as the ratio of surface to bulk conductance [27]. For a channel with an a×b rectangular cross-section, $Du=2K_{s}\left(a+b\right)/\left(ab\sigma \right)$, where $K_{s}$ is the surface conductance and σ is the electrolyte conductivity. In our case, σ=1.45 mS/m, *a* = 80 μm, and *b* = 50 μm. We assume a typical value of $K_{s}=1$ nS, as reported in Refs. [28,30], which yields Du≈0.065. On this basis, the contribution of surface conduction was neglected in the estimation of the theoretical channel resistance. Nevertheless, we acknowledge that surface conduction along the channel walls may introduce a correction below 10%.

### 3.3. Electrophoresis Experiments

**Figure 5:**
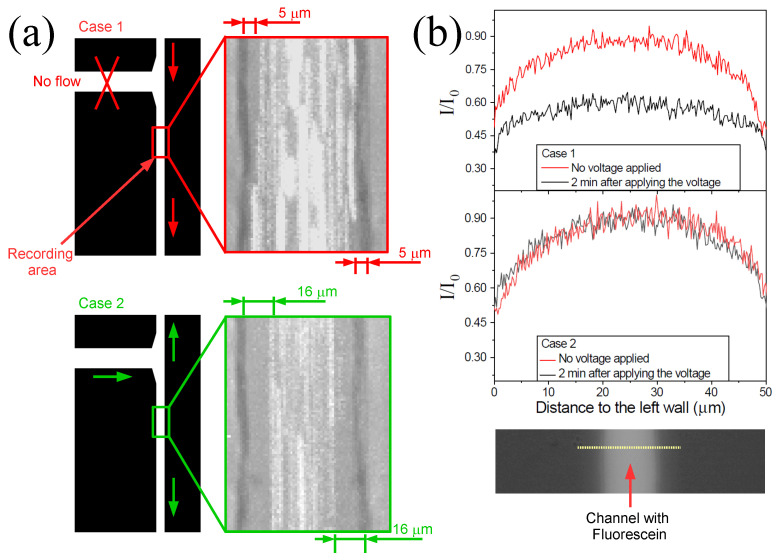
(**a**) Stack of video frames recorded during the experiments designed to measure wall separation under two different conditions: (case 1) with the flow direction promoting an increase in H^+^ concentration, and (case 2) with the presence of H^+^ ions minimized by using the configuration described above. Particle velocity at the center of the channel was 57 mm/s in both cases. (**b**) Normalized intensity of the fluorescent dye emission along the channel width, before and after applying the voltage, for the two different cases. The intensity was measured along the dashed line shown in the snapshot. Intensity data were normalized using the maximum value.

To illustrate how an increase in H^+^ concentration affects the cross-stream migration in electrophoresis experiments, we conducted an experiment to measure the distance to the wall of 3 μm particles under two different conditions. The observation area was located 5 mm downstream from the intersection, within the channel connecting the intersection to inlet 3 (see Figure 5a). In one case, labeled 1, the flow direction through the channel promotes an increase in H^+^ concentration. In the other case, case 2, the presence of H^+^ ions is minimized by employing the configuration described previously. An image of the observation area is shown in Figure 5a for both cases. The video frames recorded during the experiments were stacked using ImageJ (https://imagej.net/ij/, accessed on 8 January 2026). The presence of the 3 μm particles is indicated by their white traces. To better highlight the contrast between the two scenarios, the results for case 1 are shown at the top, while those for case 2 are presented at the bottom. In both cases, the flow velocity at the center of the channel was measured to be 57 mm/s. The formation of a depletion zone is observed in both configurations. This observation confirms the occurrence of particle drift perpendicular to the electrophoretic motion in both cases. Nonetheless, it is worth noting that the magnitude of this drift is greater in case 2, where the depletion zone extends up to 16 μm from the wall. In contrast, in case 1, the depletion zone is nearly three times narrower. The depletion zone is defined as the distance from the wall within which 95 % of the particle is located. These experiments were repeated three times with microfluidic channels fabricated anew to confirm reproducibility and we obtained that the variability in the extension of the depletion zone is less than 7%. Wall separation in electrophoresis experiments has been extensively studied under a wide range of conditions. For low Reynolds numbers and AC fields, the separation is well explained by the electroosmotic flows which arises as a consequence of concentration polarization around the particles [8,9,31]. We refer to these flows as concentration polarization electroosmosis (CPEO) [32,33]. However, under the experimental conditions examined in this study—including DC fields and Reynolds numbers around 1—there is no clear consensus on the origin of the interaction with the wall, and it remains an active area of research [21,34]. Regardless of its origin, the clarification of which lies beyond the scope of this work, the results presented here underscore the importance of preventing an increase in H^+^ concentration during electrophoresis experiments, as it clearly impacts the outcomes. To demonstrate that the pH within the channel varies depending on the configuration used, we measured the light intensity (fluorescein brightness) along the channel width. Figure 5b shows the normalized intensity values for the two configurations described in Figure 5a (case 1 and case 2), measured along the dashed line shown in the snapshot below the graphs, both before and two minutes after applying the voltage. Only the intensity values within the channel are shown. The curves were normalized using the maximum intensity value. In case 1, after two minutes, the pH exhibited a noticeable change, characterized by a decrease in intensity. In contrast, no change was observed in case 2, confirming that this configuration effectively prevents the entry of $H^{+}$ ions.

## 4. Conclusions

We have proposed a simple microfluidic device to mitigate the impact of Faradaic currents on DC electrophoresis experiments. We have investigated the interplay between the advection and electromigration of H^+^ ions in a microfluidic PDMS device under DC electric fields and Reynolds numbers around 1. Our results show that the advection can effectively limit the accumulation of H^+^ ions in the channel when its velocity exceeds the electromigration velocity (∼28 mm/s). This balance directly impacts the local pH and conductivity, as demonstrated by changes in electrical resistance of the channel and fluorescence intensity of a pH-sensitive dye. The separation of channel walls of 3 μm particles was observed to be significantly stronger when H^+^ ions were minimized, highlighting the critical role of acidification in cross-stream particle migration in electrophoresis experiments. While the exact origin of the particle-wall separation under these conditions remains unresolved, our findings emphasize the importance of controlling the effect of Faradaic reactions to ensure accurate and reproducible electrophoresis experiments.

## Data Availability

Data supporting this study are available from the authors upon request.
